# Towards an appropriate framework to facilitate responsible inclusion of pregnant women in drug development programs

**DOI:** 10.1186/s13063-018-2495-9

**Published:** 2018-02-20

**Authors:** Kit C. B. Roes, Indira S. E. van der Zande, Maarten van Smeden, Rieke van der Graaf

**Affiliations:** 1 Department of Biostatistics and Research Support, Julius Center for Health Sciences and Primary Care, University of Utrecht, University Medical Center Utrecht, Utrecht, the Netherlands; 2 Department of Medical Humanities, Julius Center for Health Sciences and Primary Care, University of Utrecht, University Medical Center Utrecht, P.O. box 85500, 3508 GA Utrecht, the Netherlands; 3 Department of Clinical Epidemiology, Leiden University Medical Center, Leiden, the Netherlands

**Keywords:** Research design, Pregnant women, Methodology, Research ethics, Adaptive design, Drug development

## Abstract

Evidence-based treatment for pregnant women will ultimately require research conducted in the population of pregnant women. Currently, few scholars have addressed the issue of responsible inclusion of pregnant women in drug research. Because of additional risks associated with including pregnant women in drug research and the altered ways in which drugs are processed by the pregnant body, pregnant women cannot be treated as an ordinary subgroup in the various phases of drug development. Instead, responsible inclusion of pregnant women requires careful design and planning of research for pregnant women specifically. Knowledge about these aspects is virtually nonexistent.

In this article, we present a practical framework for the responsible inclusion of pregnant women in drug development. We suggest that the framework consists of using a question-based approach with five key questions in combination with three prerequisites which should be addressed when considering inclusion of pregnant women in drug research. The five questions are:

A. *Can we consider the drug safe (enough) for first exposure in pregnant women and fetuses?*
B. *In which dose range (potentially depending on gestational age) can the drug be considered to remain safe in pregnant women?*
C. *At what dose (regimen, within the range considered safe) can we expect efficacy in pregnant women?*
D. *Can efficacy be confirmed at the target dose, either similar to the initial population or different?*
E. *Can clinical safety be confirmed at a sufficiently acceptable level at the target dose for pregnant women and fetuses, so as to conclude a positive benefit–risk ratio?*

Combining questions and prerequisites leads to a scheme for appropriate timing of responsible inclusion of pregnant women in drug research. Accordingly, we explore several research design options for including pregnant women in drug trials that are feasible within the framework. Ultimately, the framework may lead to (i) earlier inclusion of pregnant women in drug development, (ii) ensuring that key prerequisites, such as proper dosing, are addressed before more substantial numbers of pregnant women are included in trials, and (iii) optimal use of safety and efficacy data from the initial (nonpregnant) population throughout the drug development process.

## Background

Over the past decades, bioethicists, pharmacologists, regulators, and researchers have called attention to the inclusion of pregnant women in clinical research in order to improve the evidence base underlying maternal and fetal health [1–5]. During pregnancy, women may suffer from serious acute and chronic obstetric or non-obstetric illnesses that require drug treatment in the interest of both the mother and the fetus. Examples of illnesses are mental disorders, hypertension, asthma, diabetes, cancer, and autoimmune disorders [1, 2]. It is estimated that 84–99% of women take medications during pregnancy, for which there are no substantial data on safety, efficacy, or fetal risk [6–9]. The lack of a sound evidence base leads to suboptimal care or even undertreatment of pregnant women.

To bridge the knowledge gap regarding safe and effective drug use in pregnant women, various stakeholders have taken up the challenge of inclusion. Already in 1994, the Institute of Medicine stated that pregnant women are presumed to be eligible for participation in clinical research, a view that was later endorsed by others [1, 4, 10]. In 2009, the Second Wave Initiative was launched, a collaborative academic initiative to find ethically and scientifically responsible means to increase the knowledge base for the treatment of pregnant women with medical illness [1, 11]. Additionally, the United States Food and Drug Administration (FDA) recently replaced its traditional pregnancy categories for drug use in pregnant women by the Pregnancy and Lactation Labeling Rule (PLLR, Final Rule), which is expected to provide further incentives for the development and conduction of clinical research in pregnant women [12]. Despite these attempts to respond to the call for inclusion, the underrepresentation and exclusion of pregnant women from clinical research remains common practice [5, 13]. There are various reasons for the continuing status quo, such as a fear of harming the fetus, numerous liability concerns, and the question whether pregnant women would be willing to participate even if they were found to be eligible [14–16].

The general considerations for clinical trials, as defined by the International Conference of Harmonisation guidance (ICH E8), also take a cautious approach regarding the inclusion of pregnant women in clinical trials [17]. The guideline strongly advises against inclusion if a new drug is not intended for use in pregnancy. Moreover, one unresolved, yet very essential, element is the challenge of designing studies that warrant responsible inclusion of pregnant women in drug research. Pregnancy can alter the ways that drugs act on and are processed by the body in a fashion difficult to predict from the pharmacokinetics (PK) and pharmacodynamics (PD) in men and nonpregnant women. Answering PK and PD questions for pregnant women therefore requires the development of different or new research designs for drug research [1, 18, 19].

Francoise Baylis and colleagues have been the first to address ethically responsible inclusion of pregnant women in drug research, by proposing two alternative approaches. Baylis and colleagues argue for a particular type of routine inclusion of pregnant women in clinical studies of drug safety and effectiveness, except when there are compelling scientific or ethical reasons to exclude pregnant women [3, 20]. They start from the assumption that it is ethically preferable to (a) expose a limited number of pregnant women and their fetuses to a new drug in very well-controlled conditions at an early stage first, compared to (b) not doing so, and instead relying on information from exposure of large numbers of pregnant women and fetuses in less well-controlled conditions after post-marketing authorization. Although we do not underwrite routine inclusion, we sympathize with the work of Baylis and colleagues and their attempt to take on the challenge of research design for pregnant women. However, their proposal can be further strengthened. What is particularly missing in the current discussion on the inclusion of pregnant women in drug trials, and what we will address in this article, are (i) thoughts about the level of evidence needed from pregnant women to ensure safe and effective drug use, (ii) guidance to help decide on appropriate timing (in the development course of a drug), if at all, for the inclusion of pregnant women in drug trials, and (iii) a more extensive exploration of research designs that can facilitate inclusion.

The aim of our article is to present a practical framework for responsible inclusion of pregnant women in drug development. Our article further suggests directions for trial design that support safe and efficient inclusion of pregnant women in different stages of drug development. First, we introduce the practical framework, which consists of a question-based approach in combination with three prerequisites. The framework provides a reasonable and efficient method for the design of a drug development program in the form of a scheme [21, 22]. Second, we evaluate the proposal of Baylis and colleagues in light of our framework. Third, we extend the discussion beyond the scope of phase I trials and use the framework to explore practical suggestions for key (statistical) design features of clinical studies. As such, we address potential safety concerns and thus place the contribution of Baylis and colleagues in a broader context. Finally, we discuss the practical implications of our proposal. Our scope encompasses the development of new drugs for non-obstetric illnesses. Note, we do not attempt to provide a conclusive answer regarding specific trials, but instead aim to prompt a methodological discussion on the inclusion of pregnant women in drug trials.

### Framework: question-based approach and prerequisites

Traditionally, the timing and sequential order of clinical research studies for new drugs comprises four phases: first exposure in humans and primary safety (phase I), establishing the efficacious and safe dose (phase II), confirming efficacy and safety in a broader population (phase III) and additional studies post-market authorization (phase IV) (see also [17]). The current paradigm in drug development splits the pre-marketing drug development process in roughly two larger phases: “Learning” and “Confirming” [23]. Given the (unknown) risks and possible serious consequences for pregnant women and fetuses, including them as an ordinary subgroup in the regular phases is often unwarranted. Instead, an approach is needed that ensures an adequate level of evidence of safety and efficacy for inclusion of pregnant women. We propose a question-based approach.

Similar to the traditional four-phase set-up, a question-based approach assumes that a clinical drug development program ultimately aims to answer pertinent questions about a new drug, from fundamentals about the mechanism of action and its effects in the human body, up to clinical efficacy and safety [21, 22]. A question-based approach specifically acknowledges that different types of questions may require different clinical research designs, and that the right order of addressing the questions may increase the relevance and safety of the information and efficiency of decision-making [21, 22]. As such, the principle starting point is that these questions do not differ between the initial population (here and hereafter referring to: men and *nonpregnant* women, or nonpregnant women only) and pregnant women, but essentially that pregnancy adds complexity and additional safety concerns. A question-based approach can clarify in which phase which question should be answered, before pregnant women can be enrolled. An important advantage of looking at the situation from a question-based perspective, rather than (only) phase I–IV based, is that the research question is made explicit and all options to obtain an appropriate answer can be considered. This may very well prevent unnecessary clinical studies in pregnant women, as we will argue below.

Applying a question-based approach to the inclusion of pregnant women in clinical studies for new drug treatments, we assume that a complete development plan for a specific drug is already foreseen in the initial population. Since we are particularly concerned with clinical efficacy and safety, we suggest that the key questions to (potentially) address for pregnant women are:

A. *Can we consider the drug safe (enough) for first exposure in pregnant women and fetuses?*
B. *In which dose range (potentially depending on gestational age) can the drug be considered to remain safe in pregnant women?*
C. *At what dose (regimen, within the range considered safe) can we expect efficacy in pregnant women?*
D. *Can efficacy be confirmed at the target dose, either similar to the initial population or different?*
E. *Can clinical safety be confirmed at a sufficiently acceptable level at the target dose for pregnant women and fetuses, so as to conclude a positive risk–benefit ratio?*

Questions A to C fall under “Learning”. To arrive at a negative answer, usually in terms of safety, research in the initial population or even preclinical research alone may suffice. Hence, answering questions A to C may not always necessitate clinical research in pregnant women. Questions D and E are confirmatory, and would need research in the target population of pregnant women. Naturally, different questions can be answered within the same study. To illustrate, most clinical studies will address both efficacy and safety questions. Combining learning and confirming questions within the same study is more controversial [24], but can be realized with an adaptive clinical trial design [25]. So the questions that are specifically targeting pregnant women could be answered with a separate trial, but also with a trial in which pregnant women constitute a subgroup among the initial population.

Generally agreed-upon prerequisites for clinical trials in pregnant women are threefold [26]. First, adequate preclinical and early clinical data pertinent to pregnancy must be available before first exposure in pregnant women. This data would ideally include PK data from nonpregnant women and animal data including data from pregnant animals, as well as preclinical and in vitro models of placenta transfer, and, if possible, of placental transport, metabolism, and endocrine function [26]. Second, clinical exposure in pregnant women should preferably start once basic clinical safety data in the initial population is known and can be used to assess potential risks for pregnant women [3, 20]. Third, clinical efficacy should preferably be established to a sufficient extent in the initial population, before exposing larger numbers of pregnant women. That way, one would avoid exposure to a potentially noneffective drug. Combining a question-based approach in which the five clinical questions are addressed with the three prerequisites leads to a scheme with acceptable options for including pregnant women in a drug development program (Table 1).

**Table 1:**
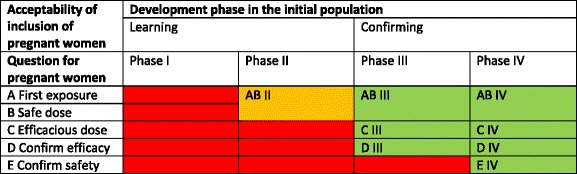
Proposed admissible timing scheme for question-based inclusion of pregnant women relative to the different phases of drug development in the initial population

In Table 1, it is assumed that for a clinical trial addressing confirmatory questions D and E, questions A to C have already been answered adequately (by means of a clinical trial or otherwise). Furthermore, gestational age is likely to impact the PK of drugs [26], and should thus be included in all design considerations. It is worth noting that inclusion of pregnant women at different gestational ages may impact the total sample size, depending on the disease and duration of exposure. Additionally, we assume that follow-up of fetuses and children is part of every trial with pregnant women.

The framework (i.e., the scheme resulting from the combination of a question-based approach and the prerequisites), enables us to more systematically assess potential design options for the inclusion of pregnant women in drug trials.

### Applying the framework to Baylis’ and Halperin’s proposal

Baylis and Halperin have considered two modes of conducting phase I trials in pregnant women during phase II and phase III trials in the initial population [20]. One of their proposed alternatives is to run a separate phase I trial in pregnant women in parallel with a phase III in the general population. The other proposal is to embed the phase I trial features for pregnant women (including intensive safety monitoring) within a late phase II or phase III trial. As Baylis and Halperin explicate, the primary advantage of timing a phase I in pregnant women during a phase III in the initial population is that efficacy and safety data can be evaluated prior or concurrently to the initial population. Moreover, information from earlier drug trials can better inform researchers about potential risks and benefits of that same drug in pregnant women. As such, the phase I trial in pregnant women (separate or embedded) may avoid unnecessary testing of drugs in pregnant women that are proven insufficiently safe in the initial population.

In our proposed framework, the two designs of Baylis and Halperin presumably aim to answer question A (safety) and potentially question B (effective dose). It appears that Baylis and Halperin assume that question A (is the drug safe enough for first exposure in pregnant women) was answered positively, based on the preclinical and clinical research in the initial population. However, it is not clear how an appropriate dose range for pregnant women is subsequently achieved (question B). If a phase I trial with pregnant women is embedded in a phase III trial, evidence of efficacy and clinical safety will most likely be generated in pregnant women (questions C to E). This could happen in the sense of evaluating consistency of efficacy and safety with the initial population, but this is not specifically mentioned. Additionally, Baylis and Halperin do not address (statistical) design features that may provide further safeguards for pregnant women and fetuses. In the following, we will extend the ideas of Baylis and Halperin by addressing ideas on clinical research design options in more detail.

### Applying the framework further: extended design options and considerations for trials

We will now put our framework (Table 1) into context. We will discuss each of the five questions, address the important issue of when to answer these questions and provide practical guidance and examples of research designs wherever possible.

#### Design options during phase II, addressing questions A and B (AB II)

- *Question A: can we consider the drug safe (enough) for first exposure in pregnant women and fetuses?*
- *Question B: in which dose range (potentially depending on gestational age) can it be considered to remain safe in pregnant women?*

In most cases, it is too early to address questions A and B in pregnant women in parallel with a phase II trial in the initial population, because phase I data from the initial population is insufficiently informative on the appropriate (safe and effective) dose for pregnant women. Moreover, phase II safety data from the initial population (typically laboratory data and adverse experiences) is missing. Nevertheless, as it is crucial to determine appropriate dosing for pregnant women in light of their specific physiology, in some cases we could imagine careful first exposure of pregnant women at this stage. For question A, the preclinical and phase I data in the initial population might, in some cases, be considered adequate, if only a limited dose range in pregnant women is evaluated subsequently during phase II. This limited dose range can provide important PK information on dosing in pregnant women, possibly preventing safety risks later on in the drug development program. Question B could (partially) be answered early (in parallel with phase II in the initial population), by gathering data in pregnant women at (very) low doses of the new drug, based on extrapolation from exposure in the initial population (e.g., obtained in phase I) to exposure in pregnant women, possibly at different gestational ages.

A stepwise adaptive trial could be considered to support model-based extrapolation to a proper dose in pregnant women for a future trial. When planned after the phase I trial in the initial population, essential human PK properties are known (linear or nonlinear kinetics, dose exposure relations), as well as some PD. A (small) trial could be designed involving pregnant women of different gestational ages. The objective would be to optimize an extrapolation model of existing PK to pregnant women, to enable the selection of an appropriate dose for therapeutic trials. First exposure would be at (very) low doses, and would assess, and subsequently carefully increase, PK. This can be based on a translational model from the available and new PK data, by “matching” the exposure as observed in the initial phase I PK. As such, the trial can be used to optimize the extrapolation model of PK to pregnant women, while remaining in a sufficiently safe dose range. Question B will thus be answered, albeit approximately, resulting in estimated effective doses for future trials. Furthermore, the risk of exposing pregnant women to a potentially unsafe high dose at a later stage, which exists in absence of the extrapolation data, is reduced.

#### Design options during phase III, addressing questions A and B (AB III)

- *Question A: can we consider the drug safe (enough) for first exposure in pregnant women and fetuses?*
- *Question B: in which dose range (potentially depending on gestational age) can it be considered to remain safe in pregnant women?*

Answering questions A and B for pregnant women concurrent with a phase III trial in our framework is in line with Baylis and Halperin’s proposal for a phase I study in pregnant women in parallel to a phase III study in the initial population.

A phase I study in pregnant women in parallel with a phase III study in the initial population would entail a more traditional phase I study, including escalating doses which are guided by PK and safety considerations. Dose escalation then needs to be established up to the level where exposure is expected to be therapeutic in pregnant women. Pregnant women of different gestational ages would need to be included. Moreover, the above-described PK extrapolation approach is needed here as well, in order to determine an appropriate and safe dose escalation scheme. Examples of these types of studies and timing can typically be found in drug development for infectious diseases (see clinicaltrials.gov). A practical illustration is the phase I PK and safety study of a ledipasvir/sofosbuvir (LDV/SOF) fixed-dose combination, in pregnant women with a chronic hepatitis C virus (HCV) infection (results not reported yet) [27]. LDV/SOF are new directly acting antiviral drugs for the treatment of HCV that are proven effective and well tolerated. Preclinical evaluations in animal models indicate safe administration during pregnancy. This single-arm, open-label phase I study in 15 pregnant women evaluates the safety and PK of antenatal LDV/SOF treatment for 12 weeks during the second and third trimester. Therapy is initiated at approximately 24 weeks of gestation. The study aims to determine: (i) if the PK of LDV and SOF is similar in pregnant women as compared to nonpregnant women, (ii) if the viral response to LDV/SOF treatment in pregnant women is similar to that observed in nonpregnant women, and (iii) if any initial maternal or neonatal safety concerns are detected with antenatal LDV/SOF administration.

#### Design options during phase III, addressing questions C and D (C III to D III)

- *Question C: at what dose (regimen, within the range considered safe) can we expect efficacy in pregnant women?*
- *Question D: can efficacy be confirmed at the target dose, either similar to the initial population or different?*

Answering questions C and D in our framework is in line with Baylis and Halperin’s earlier explained proposal to embed the phase I trial for pregnant women in the phase III trial for the initial population. But in contrast to Baylis and Halperin, we argue that answering questions C and D for pregnant women extends well beyond embedding a phase I trial in a phase III trial. In order to provide an answer, it is not sufficient to generate phase I data for pregnant women within the context of the phase III trial, more pertinent data is needed as well. For example, data on clinical efficacy, or safety over a relevant treatment period given the disease. Moreover, pregnant women, and to the extent possible their fetuses, need to be monitored intensively from a safety perspective in all cases. Preferably in line with phase I/first-in-human studies, even if it is not the first exposure.

Furthermore, before considering enrolling pregnant women in a phase III trial and reflecting on the potential objectives for doing so, it is worthwhile considering additional prerequisites that would have to be taken into account. First, a minimum prerequisite for considering the new treatment efficacious in pregnant women is that efficacy is demonstrated in the initial population. Second, when enrolling pregnant women in a phase III trial simultaneously with the initial population, a prerequisite is that the safety of the dose(s) used in pregnant women should be optimal. Hence, the scenario of addressing questions C and D concurrently with the regular phase III trial would require some kind of phase I study in pregnant women at an earlier stage (so that question B is addressed specifically for pregnant women), or reliable extrapolation data based on data from the initial population.

There are a number of design considerations with egard to addressing questions C and D in a phase III trial. One important consideration is that the subgroup of pregnant women would typically not be large enough to stand on its own. Following our proposed prerequisites, exposing larger numbers of pregnant women would only be justified if efficacy is sufficiently established in the initial population. Additional safeguards can be built in, based on preplanned interim analyses. In a phase III trial in which pregnant women would enroll from the start, interim analyses would include early stopping for safety or efficacy reasons. With respect to efficacy, it is possible to use interim analyses to analyze whether efficacy in pregnant women is (considerably) less promising as compared to the initial population. This would allow early stopping of the group of pregnant women, thus avoiding risks where there might not be benefit.

Alternatively, more intensive monitoring at the individual subject level can provide the necessary protection for participating pregnant women and fetuses. A recent example which illustrates how monitoring can provide a safety safeguard is the upcoming safety and exploratory efficacy study of an investigational anti-influenza immune plasma for the treatment of influenza, where adults, children and pregnant women are to be simultaneously included [28]. The study will assess the safety, efficacy, and PK of anti-influenza plasma, as a treatment for strains that are resistant to current antiviral treatment, in patients at risk for severe diseases. A total of 100 subjects will be randomized in a 1:1 ratio, to receive either 2 units (or pediatric equivalent) of anti-influenza immune plasma in addition to standard care, or standard care alone. In this case, additional safeguards for safety stem from the fact that subjects are hospitalized with influenza and therefore intensively monitored.

**Fig. 1:**
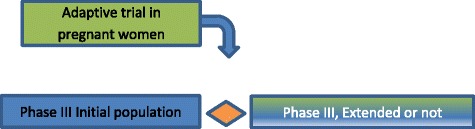
Adaptive phase III trial with interim extension of recruitment based on adaptive trial in pregnant women

Finally, when there is substantial residual uncertainty at the start of a phase III trial, an adaptive approach is worth considering. In this case, the phase III trial can start without including pregnant women. An interim decision can be made in phase III to extend recruitment to pregnant women (see Fig. 1), based on results of an adaptive trial in pregnant women to arrive at a proper and safe dose (following the adaptive design as described above). The statistical approach for such a design could be based on the methodology of Bauer and Köhne, which can be applied to the group of nonpregnant women to establish confirmatory evidence of efficacy on the combined date before and after the adaptation [25]. The subgroup of pregnant women can be evaluated separately (albeit with limited power), and consistency of treatment effect estimates between pregnant and nonpregnant women can be assessed similar to other subgroup evaluations [29].

#### Design options during phase IV, addressing all questions (AB IV to E IV)

- *Question A: can we consider the drug safe (enough) for first exposure in pregnant women and fetuses?*
- *Question B: in which dose range (potentially depending on gestational age) can it be considered to remain safe in pregnant women?*
- *Question C: at what dose (regimen, within the range considered safe) can we expect efficacy in pregnant women?*
- *Question D: can efficacy be confirmed at the target dose, either similar to the initial population or different?*
- *Question E: can clinical safety be confirmed at a sufficiently acceptable level at the target dose for pregnant women as well as their fetus, so as to conclude a positive benefit–risk ratio?*

Addressing questions A to E in phase IV would mean that a new treatment is already marketed, before pregnant women have participated in clinical trials. At this point, efficacy and safety in the initial population are sufficiently established. If questions for pregnant women are answered at all, they are generally answered through case studies of pregnant women using the drugs off-label. Currently, off-label use is the most common situation. However, there are many challenges that require similar safeguards, as in the clinical trial designs that were introduced above. For one thing, a proper dose for pregnant women needs to be established. This requires a phase I-type trial and a careful stepwise evaluation of safety, including nonclinical safety investigations. As the opportunity to include pregnant women in a phase III trial in the initial population is currently not used, in most cases this means that a separate efficacy and safety study in pregnant women is (still) needed in order to address questions A and B.

Evidently, delay is a negative consequence of requiring a phase I study before allowing pregnant women in phase IV studies. And if we do not require a phase I study but depend on observational data instead, we are still faced with a similar timeframe, since it may take years before sufficient observational data is collected [30]. Because of these delay issues, we concede that there are cases in which pregnant women could be included in phase IV trials, even when questions A to E are not answered in trials for pregnant women. Depending on the general safety profile and the results of a phase I-type study in pregnant women (PK and dosing), close monitoring based on an observational registry in which data is systematically gathered might suffice.

## Discussion

Including pregnant women in regular drug development programs is unwarranted due to the often unknown risks and potential serious consequences for pregnant women and fetuses. Pregnant women cannot be considered as an “ordinary subgroup” for which the traditional four-phases approach towards drug development could apply. Instead, we proposed a practical framework for planning the inclusion of pregnant women in drug development, in the form of a question-based approach in combination with prerequisites. Specifically, we formulated five key clinical research questions and complemented the questions with three generally agreed-upon prerequisites in order to determine concurrently with what phase of the traditional development program the questions should be answered for pregnant women. Based on the combination of questions and prerequisites, a scheme for responsible inclusion of pregnant women in drug trials could be drafted (Table 1). Accordingly, we argued that question A and B first need to be answered positively for pregnant women, to establish proper safe dosing as a key prerequisite (parallel/embedded in a phase II or phase III trial in the initial population), before question C to E can be answered by including pregnant women (parallel or embedded in a phase III or a phase IV trial in the initial population). Consequentially, we proposed that in most cases, a phase I trial in which data on drug safety and drug dose range is collected, should always be conducted before including pregnant women in later phases. By indicating which information needs to be addressed at what time, we demonstrated different possibilities of responsibly including pregnant women at an earlier time in the drug development process.

The planning of including pregnant women in drug programs is a relatively unexplored field and there are a number of additional aspects that need exploration in order to determine the viability of our framework. One such aspect involves the monitoring of safety and follow-up of pregnant women and fetuses, which should have a place in any scenario. Presently, there are requirements for monitoring and, if possible, follow-up [8], but these requirements seem insufficient because they do not stipulate the method for monitoring or follow-up. Some countries have experience with compulsory pregnancy registries (for example the Swedish Medical Birth Register), which enables the collection of large numbers of maternal medication data. At the same time, such registries have their own challenges. Further research into adequate monitoring and follow-up is necessary, but is outside the scope of our article.

It could be argued that our framework, which specifically requires the establishment of safety and dose range in a phase I trial, may delay inclusion of pregnant women in drug research. Nevertheless, delay could be partly avoided, for example if preclinical data and extrapolation of phase I data from the initial population would allow exposure at low doses in pregnant women. Moreover, our article actually emphasizes the different options of including pregnant women at an *earlier* phase, in order to increase the possibilities to conduct research in pregnant women. By indicating the appropriate time at which inclusion of pregnant women can be safe and therefore acceptable, we remove design barriers that have hitherto hindered inclusion of pregnant women in drug trials. We challenge the current underrepresentation and we support the idea that including a smaller group of pregnant women in a well-controlled setting is preferable to exposing the whole population of pregnant women to unknown risks. We hope that our discussion on the appropriate timing and the different design options for the responsible inclusion of pregnant women will ultimately contribute to the development of specific trial designs for pregnant women.

### Limitations

This study has some limitations. First, the current exploration does not include a full practical application and the actual proof would be a fully developed protocol and evidence of feasibility through adequate recruitment and conduct. Second, our proposal assumes that funding agencies and manufacturers are willing to include pregnant women in clinical research. Further research should explore if they are indeed willing to recruit pregnant women for our proposed research designs. Third, irrespective of the design, intensive monitoring and long-term follow-up of women, fetuses, and newborns is essential. While we did not extensively address monitoring and follow-up, the present limitations in physiologic and medical follow-up of the fetuses and newborns may still be a serious hurdle to the inclusion of pregnant women, which cannot be overcome by clinical trial methodology alone.

## Conclusions

In this article, we have argued that a practical framework for the inclusion of pregnant women in drug research could consist of the combination of a question-based approach with prerequisites for drug development for pregnant women. The framework includes a scheme for the safe and appropriate timing of inclusion of pregnant women, concurrent with the regular drug development program. Ultimately, our framework may lead to (i) earlier inclusion of pregnant women in drug development, (ii) ensuring that key prerequisites such as proper dosing, are addressed before more substantial numbers of pregnant women are included in trials, and (iii) optimal use of safety and efficacy data from the initial (nonpregnant) population throughout the development program. By indicating the appropriate time at which inclusion of pregnant women can be safe and therefore acceptable, we aim to remove design barriers that have hindered inclusion of pregnant women in drug trials. We challenge the current underrepresentation and we support the idea that including a smaller group of pregnant women in a well-controlled setting is preferable to exposing the whole population of pregnant women to unknown risks. We further hope that our discussion will contribute to the development of specific trial designs for pregnant women, which is essential in order to increase the evidence base for pregnant women and fetuses.

## Abbreviations

FDA: Unites States Food and Drug Administration
HCV: hepatitis C virus
LDV: ledipasvir
PD: Pharmacodynamics
PK: Pharmacokinetics
PLLR: Pregnancy and Lactation Labeling Rule, SOF, sofosbuvir
